# Effects of Wax Molecular Weight Distribution and Branching on Moisture Sensitivity of Asphalt Binders

**DOI:** 10.3390/ma15124206

**Published:** 2022-06-14

**Authors:** Wenqi Wang, Azuo Nili, Ali Rahman, Xu Chen

**Affiliations:** 1 School of Architecture and Civil Engineering, Xihua University, Chengdu 610039, China; 0120030057@mail.xhu.edu.cn; 2 School of Civil Engineering, Southwest Jiaotong University, Chengdu 610031, China; azuo.nili@my.swjtu.edu.cn (A.N.); arahman@swjtu.edu.cn (A.R.); 3 Highway Engineering Key Laboratory of Sichuan Province, Southwest Jiaotong University, Chengdu 610031, China

**Keywords:** molecular weight distribution, branching of wax, asphaltene, moisture sensitivity, surface free-energy, binder bond strength

## Abstract

Wax is an important factor that affects the durability of asphalt binder. In order to understand the molecular weight distribution and branching of wax on the moisture sensitivity of asphalt binder, pure wax-doped asphalt binders are prepared and the performance of model asphalt binders are evaluated by surface free-energy (SFE) and binder bond strength (BBS) tests. In addition, asphaltene is regarded as an additive in this study. The results show that the addition of eicosane, triacontane, squalane and asphaltene can reduce the moisture sensitivity of asphalt, but not necessarily improve its moisture-induced damage resistance. The physical hardening effect of high-wax asphalt and its model asphalt is stronger than that of the corresponding low-wax asphalt and its model asphalt, and its moisture sensitivity is weaker than that of the low-wax asphalt. For all the model asphalts, there is a good correlation between the cohesion work, cohesion POTS (pull-off tensile strength), POTS ratio (the BBS moisture sensitivity index) and ER (the SFE moisture sensitivity index). When using the BBS test to characterize the moisture sensitivity of high-wax asphalt, it is recommended to leave the sample for some time until it is physically hardened and stable.

## 1. Introduction

**Figure 1:**
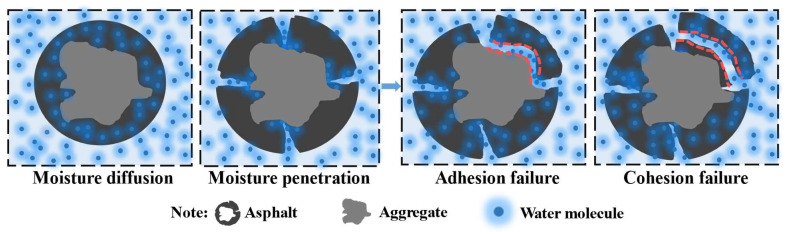
Moisture-induced damage process diagram of asphalt pavement. “Reprinted with permission from Ref. [4]”.

The strong moisture sensitivity of asphalt mixture is one of the main reasons for early distresses in asphalt pavement, such as grouting, spalling, looseness and potholes [1]. The process of moisture-induced damage to an asphalt mixture is complex, involving many reactions and theories, such as mechanics, physics, chemistry and thermodynamics [2]. It mainly includes two stages: (1) moisture transmission in asphalt mixture; (2) moisture damage on asphalt–asphalt (cohesive failure) and asphalt–aggregate (adhesion failure) interfaces; see Figure 1. The latter is regarded as the two main and direct mechanisms of moisture-induced damage on asphalt pavement [3]. Therefore, it is of great significance to study the moisture sensitivity of asphalt based on its cohesion and adhesion failure.

The wax in asphalt is a mixture of saturated n-alkanes and a small amount of isoalkanes, with carbon atom numbers ranging from 20 to 40 [5,6]. The wax content has a significant effect on the thermal sensitivity of asphalt, which is in a melting state at high temperatures and is crystallized at low temperatures [7,8,9]. Kriz et al. [10] and Isaac et al. [11] believed that wax crystallization was the main cause of thermoreversible aging. Ding et al. [12,13], Qiu et al. [14] and Zhang et al. [15] studied the influence of a variety of wax-based warm-mix additives on thermoreversible aging in asphalt, indicating that the influence of wax-based warm-mix additives on thermoreversible aging was less than that of oxidation aging, and recommended the best combination of carbon atoms for wax-based warm-mix additives. On the other hand, due to the low mixing temperature of wax-based warm-mix asphalt mixture, moisture in the asphalt mixture cannot be completely removed, resulting in a wax-based warm-mix asphalt mixture being more vulnerable to moisture than a hot-mix asphalt mixture [16]. Nakhaei et al. [17] and Habal et al. [18] studied the influence of Sasobit (wax-based warm-mix additive) on the moisture sensitivity of asphalt binders based on surface free-energy, and the results showed that the addition of Sasobit increased the surface free-energy of asphalt, but also enhanced its moisture sensitivity.

However, the existing studies only analyzed the effect of wax on the thermoreversible aging and moisture sensitivity of asphalt from a mixture point of view (mainly wax-based warm-mix additive), and had not studied it from the molecular weight distribution and branching of wax perspectives [19,20]. In order to explore this meaningful research direction, Ding et al. [21] studied the effect of the branching of wax and asphaltene on the thermoreversible aging of asphalt binder. The results showed that C_20_H_42_ could significantly aggravate the thermoreversible aging performance of asphalt binder, while C_30_H_66_ and asphaltene have no similar effect.

To sum up, most of the existing studies on the wax of asphalt only focus on the wax-based mixture side, analyzing the effect of wax on the thermoreversible aging and moisture sensitivity of asphalt, but not the effect of the molecular weight distribution and branching of wax on the moisture sensitivity of asphalt binder. In view of this, this paper uses two mature moisture sensitivity testing methods, based on the surface free-energy (SFE) test and binder bond strength (BBS) test, to study the influence of the molecular weight distribution and the branching of wax on the moisture sensitivity of asphalt binder. In addition, because asphaltene has a strong polarity, it can explain the influence of wax on thermoreversible aging from the mechanism [22,23], and it has a strong influence on the adhesion between asphalt and aggregate. Therefore, this paper also studies the effects of asphaltene on the moisture sensitivity of the asphalt binder. It provides a theoretical basis and technical support for studying the effects of the molecular weight distribution and branching of wax on the moisture sensitivity of the asphalt binder.

## 2. Materials and Experiments

### 2.1. Materials

#### 2.1.1. Asphalt

In this paper, asphalt samples A and B are selected as the base asphalt. Sample A is a low-wax asphalt and is produced in Venezuela. Sample B is a high-wax asphalt and is produced in China. The base asphalt, with a different wax content, is selected to analyze the influence of its own wax content on moisture sensitivity. The main technical indices of asphalt A and B are shown in Table 1.

#### 2.1.2. Additives

**Figure 2:**
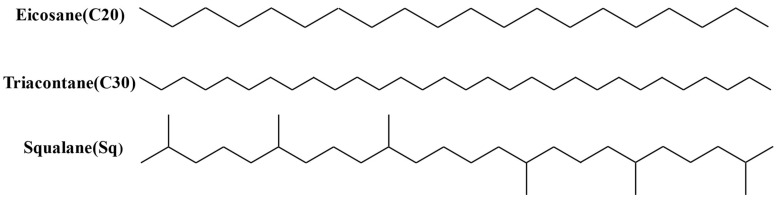
The molecular formula structure of additives.

The number of carbon atoms of wax in asphalt is between 20 and 40 [26,27]. In this paper, the additives are eicosane (C20), triacontane (C30) and squalane (Sq), and Sq is the isomer of C30. All the alkane samples mentioned above are from Benzereagen Chemical Company, Inc. The model asphalts A/B + C20, A/B + C30 and A/B + Sq can be prepared by mixing the selected alkane with asphalt A and B at 165 °C and uniformly stirring. Asphaltene (As) comes from Karamay asphalt, extracted by the solvent deasphalting (SDA) process. After the asphaltene is ground to less than 100 mesh, it is added to the base asphalt binder, and then the model asphalt, A/B + As, can be prepared. The mixing method adopted in this paper is as follows. First, heat the base asphalt to 165 °C, then slowly add the additives to the base binder. A high-mixing shear device was adopted and the mixing speed was set to 4000 rpm. The mixing time for each additive is 1 h. The above-mentioned alkane and asphaltene content are all 3%, and the following model asphalt is expressed by abbreviations. The molecular formula structure diagram and the parameters of the additive are shown in Figure 2 and Table 2.

### 2.2. Experimental Method

#### 2.2.1. BBS Test Method

**Figure 3:**
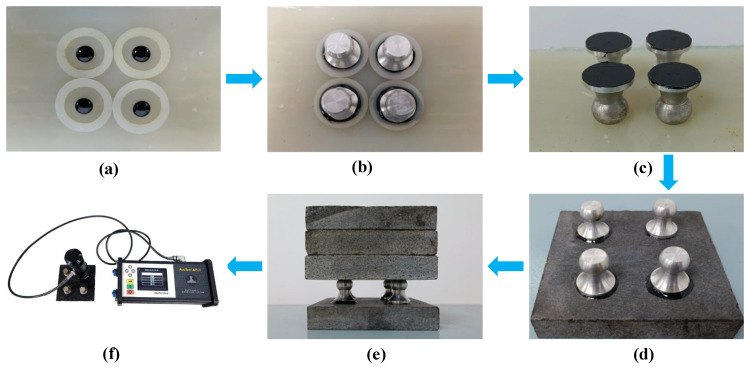
The BBS test method. (**a**) Pouring asphalt; (**b**) Sample preparation; (**c**) Attached sample (**d**) Sample on aggregate; (**e**) Loading sample (**f**) Detachment testing.

BBS tests started in the architectural coatings industry, and they can intuitively and conveniently measure the adhesive properties of materials in a short time. ASTM D4541 [28] and AASHTO TP-91 [29] have improved their testing methods to make them suitable for testing the bonding strength of the asphalt binder. In this study, the BBS tester—the pull-off adhesion tester, produced by the DeFelsko Company of the United States—is mainly composed of a test host, a drawing sleeve and a drawing head, as shown in Figure 3f. The test parameters refer to reference [4]; the pull-off tensile speed is 0.7 MPa/s and the asphalt-film thickness of the drawing head is 0.2 mm.

The forming steps of the BBS specimen are as follows: (1) cleaning slate; (2) place the asphalt, drawing head and granite plate into a 150 °C oven to heat for 30 min; (3) take the asphalt out of the oven, drop it into the silica gel pad, and immediately press the drawing head onto the top of the asphalt, as shown in Figure 3a,b, respectively; (4) after the asphalt under the drawing head is cooled, scrape off the extruded asphalt outside the drawing head, as shown in Figure 3c; (5) take out the granite slab from the oven, press the drawing head vertically onto the surface of the slab, place some heavy objects to further press the drawing head, and then place it back in the 25 °C constant temperature box for curing for some time, before testing, as shown in Figure 3d–f, respectively.

#### 2.2.2. SFE Test Method

Surface free-energy *γ* is defined as the work that the outside world needs to do when a unit surface area is generated on the surface of an object [30]. It consists of the dispersion components *γ^LW^* and polarity components *γ^AB^*, as shown in Equations (1) and (2). Because asphalt is a viscous substance, it is difficult to ensure the same dripping quality of asphalt every time, and it is easy to draw wires during dripping. Therefore, in this paper, the DSA100 contact angle meter, produced by the KRUSS company, is used to test the contact angles of the distilled water and glycerin, and to form amide with the asphalt by the sessile-drop method. Then, the contact angles are brought into the Young–Dupre equation shown in Equation (3) to indirectly calculate the surface free-energy parameters of the asphalt [31]. The SFE parameters of the three chemical reagents are shown in Table 3.

**Figure 4:**
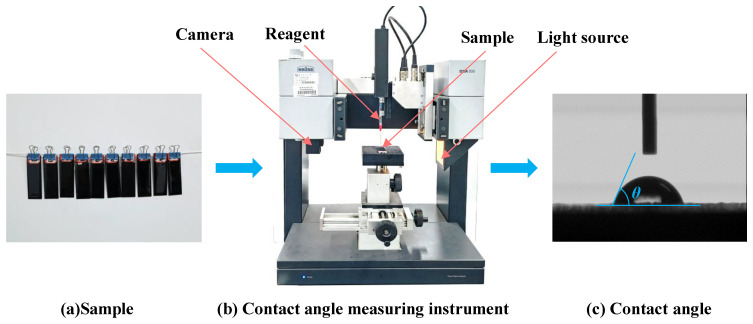
Contact angle measurement method. (**a**) Sample preparation; (**b**) Contact angle measuring instrument; (**c**) Contact angle.

The test method of the contact angle is as follows: (1) Put the asphalt sample into a 160 °C oven and heat it to a flowing state; (2) soak the cleaned glass slide in hot asphalt for 4~5 s, then hang it vertically in the oven for 10 min, so that the excess asphalt drips freely; (3) after the glass slides are cured at room temperature for a certain period of time, the contact angle tester is used to test the contact angle *θ* between the chemical reagents and the asphalt. The larger the contact angle *θ*, the more distant the mutual combination level between them, and vice versa, the closer they are, as shown in Figure 4.

(1) $$\gamma =\gamma^{LW}+\gamma^{AB}$$

(2) $$\gamma^{AB}=2\sqrt{\gamma^{+}\gamma^{-}}$$

(3) $$\frac{\left(1+COS\theta \right)\gamma_{L}}{2}=\sqrt{\gamma_{S}^{+}\gamma_{L}^{-}}+\sqrt{\gamma_{S}^{-}\gamma_{L}^{+}}+\sqrt{\gamma_{S}^{LW}\gamma_{L}^{LW}}$$

In Equations (1)–(3), *γ*, *γ^LW^* and *γ^AB^* represent surface free-energy, the dispersion component and polarity component, respectively; *γ*^+^, *γ*^−^ and *θ* represents an acid component, basic component and contact angle, respectively. The lower corner marks *S* and *L* represent the asphalt and chemical reagents, respectively.

### 2.3. Experimental Scheme

**Figure 5:**
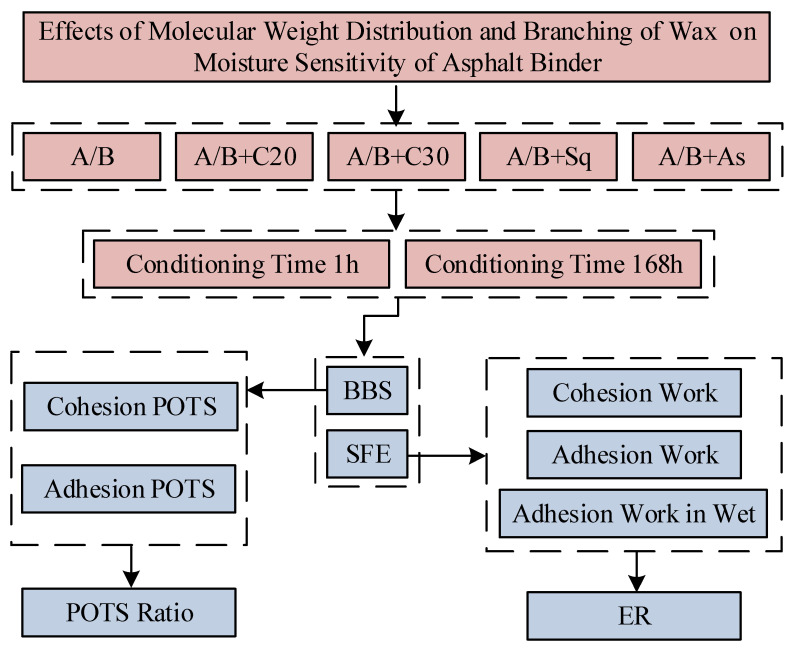
Flow chart of test scheme. Note: In this figure, cohesion POTS, adhesion POTS and the POTS ratio are the moisture sensitivity parameters and indices of the BBS test, respectively. Cohesion work, adhesion work in wet and ER are the moisture sensitivity parameters and indices of the SFE test, respectively.

In this paper, ten kinds of model asphalt (including base asphalt) are prepared by using two kinds of base asphalt and four kinds of additives, which are A/B, A/B + C20, A/B + C30, A/B + Sq and A/B + As, respectively. The BBS and SFE tests are used to test the moisture sensitivity parameters of the above ten kinds of model asphalt at room temperature and at the conditioning times of 1 h and 168 h, respectively. The parallel tests are all four groups. The specific test contents are shown in Figure 5.

## 3. Results and Discussion

The wax in asphalt has obvious thermal sensitivity. It dissolves in asphalt at high temperatures and precipitates in the form of crystals at normal and low temperatures, which makes asphalt hard. Struik [32] named this reversible reaction as physical hardening. In this paper, the degree of physical hardening (DPH) is used to describe the influence of the physical hardening of wax on the moisture sensitivity parameters of asphalt. DPH is obtained from the ratio of the moisture sensitivity parameters of the conditioning time of 1 h divided by the conditioning time of 168 h, as shown in Equation (4).

DPH = *Value(1 h)*/*Value(168 h)* (4)

In Equation (4), DPH is the abbreviation for the degree of physical hardening, and the closer its value is to 1, the smaller the physical hardening effect is, otherwise, the larger it is; *Value (1 h)* and *Value (168 h)* represent the moisture sensitivity parameter values of the conditioning times of 1 h and 168 h, respectively.

### 3.1. BBS Test

The BBS test usually shows the cohesion failure of the asphalt–asphalt interface before moisture-induced damage occurs, and the adhesion failure of the asphalt–aggregate interface after 48 h of moisture-induced damage [33]. Therefore, the BBS test, in this paper, analyzes the effects of the branching of wax and asphaltene on the cohesion pull-off tensile strength (cohesion POTS) before moisture-induced damage occurs, and the adhesion pull-off tensile strength (adhesion POTS) after moisture-induced damage. The moisture sensitivity index is calculated from it.

#### 3.1.1. Cohesion and Adhesion POTS Analysis Based on BBS

**Figure 6:**
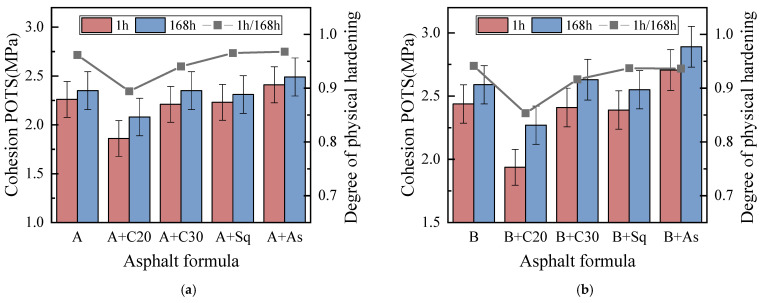
Cohesion POTS in dry conditions. (**a**) Asphalt A. (**b**) Asphalt B.

Figure 6 shows the cohesion POTS of model asphalt in a dry condition. The error bar in Figure 6 represents the variation range of four groups’ parallel test results, and the same is true of the error bar in the figure below. It can be seen from Figure 6, that the cohesion POTS of asphalt A + C20 and asphalt B + C20 is obviously lower than that of A and B, respectively, while the cohesion POTS of asphalt A + C30/Sq and asphalt B + C30/Sq has no obvious difference with the corresponding base asphalts A and B, respectively, while the cohesion POTS of asphalt A + As and asphalt B+As is higher than that of the base asphalts A and B, respectively. The results show that the effects of adding C20, C30/Sq and As into asphalt A and B on the cohesion POTS are obviously reduced, without obvious influence, and increased, respectively, which may be due to the fact that the number of carbon atoms in wax in asphalt is between 20 and 40 [34], while the carbon atoms of C20 are at a relatively small level, and C30 and Sq are at an average level. The research results show that by adding C20 and C30, similar conclusions on the thermoreversible aging performance of base asphalt can be drawn [21]. In addition, adding asphaltene increases the cohesion POTS of base asphalts A and B because asphaltene can improve its modulus and viscosity.

**Figure 7:**
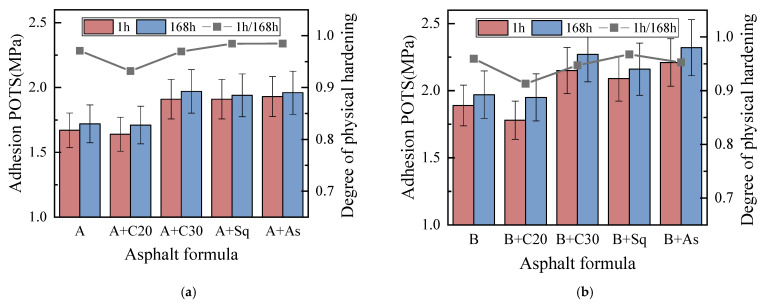
Adhesion POTS in wet conditions. (**a**) Asphalt A. (**b**) Asphalt B.

Figure 7 shows the adhesion POTS between the asphalt and aggregate after moisture-indued damage. It can be seen from Figure 7, that the adhesion POTS of asphalt A + C20 is not significantly different from that of asphalt A; the adhesion POTS of asphalt B + C20 is slightly less than that of asphalt B (within 3%); and the adhesion POTS of A + C30, B + C30, A + Sq and B + Sq are significantly higher than that of the corresponding base asphalts A and B. The results show that the influence of C20 and C30/Sq/As on the adhesion POTS of asphalt A and asphalt B has no obvious difference (asphalt B slightly decreases) and increases, and especially after asphalt B is mixed with asphaltene, it increases by about 17.5%. This is because asphaltene can increase the polarity of asphalt, thus improving the adhesion performance between the asphalt and aggregate [35].

From Figure 6 and Figure 7, it can be seen that: (1) Asphalt B and its model asphalt are larger than the corresponding asphalt A and its model asphalt, both the cohesion and adhesion POTS, indicating that the higher the wax content of the base asphalt, the greater the adhesion and cohesion POTS for itself and its model asphalt. (2) For the cohesion and adhesion POTS, the degree of physical hardening (DPH) ranges of A and B, A+C20 and B+C20, A + C30 and B + C30, A + Sq and B + Sq, A + As and B + As are about 0.94~0.97, 0.85~0.95, 0.91~0.95, 0.94~0.98 and 0.94~0.98, respectively. The results show that when C20 is added, the DPH has the greatest influence on it, followed by C30, and there is no obvious difference in base asphalt for Sq and As. This is because C20 has the smallest molecular weight and good compatibility with base asphalt. After a conditioning time of 168 h hours, C20 can precipitate from base asphalt to form crystalline wax, which hardens the asphalt and leads to the greatest DPH. However, the molecular weight of C30 is larger than C20, and the number of carbon atoms is close to the average number of carbon atoms of wax in asphalt, which makes it difficult to precipitate crystals, unlike C20. Sq is an isomer of C30. Similar to C30, asphaltene does not belong to wax (Ding et al., 2021). (3) For the adhesion and cohesion POTS, the DPH of asphalt B and its model asphalt is less than that of corresponding asphalt A and its model asphalt, respectively. The results show that the higher the wax content of base asphalt, the greater the effect of physical hardening on the adhesion and cohesion POTS of itself and model asphalt. (4) The adhesion POTS of asphalt A, asphalt B and their model asphalt are both more DPH than the corresponding cohesive POTS, indicating that the influence of DPH on the adhesion POTS is lower than the cohesive POTS. This is because the specimen will be soaked in water for 48 h before the adhesion POTS test, during which time part of physical hardening can be completed. In addition, the adhesion POTS in a wet condition is jointly determined by asphalt, aggregate and water, while the cohesion POTS is only determined by asphalt itself, which weakens the influence of physical hardening on the test results.

#### 3.1.2. Moisture Sensitivity Analysis Based on BBS

In this paper, according to AASHTO T283 [36], the ratio of mechanical strength after moisture-induced damage to that before moisture-induced damage is taken as the moisture sensitivity index of the asphalt binder. A moisture sensitivity index of the BBS test, pull-off tensile strength ratio (POTS Ratio) is put forward. The larger the value, the smaller the moisture sensitivity of asphalt. On the contrary, the larger it is, as shown in Equation (5).

POTS Ratio = Adhesion POTS/Cohesion POTS(5)

In Equation (5), Adhesion POTS represented POTS after moisture-induced damage, Cohesion POTS represented POTS before moisture-induced damage.

**Figure 8:**
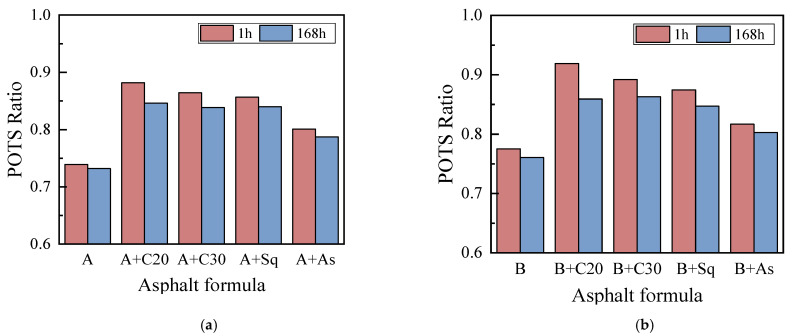
POTS ratio. (**a**) Asphalt A. (**b**) Asphalt B.

Figure 8 shows the POTS Ratio of asphalt A, B, and their model asphalt, from which we can see: (1) On the whole, adding C20, C30, Sq and asphaltene into asphalt A and B can increase their POTS Ratio and reduce moisture sensitivity, especially C20. It is worth noting that the asphalt samples with the most decrease in moisture sensitivity are not necessarily the best in moisture damage resistance. This is because the moisture sensitivity index is only the relative ratio of the POTS of the specimen before and after moisture-induced damage, and the moisture-induced damage resistance performance is also related to the absolute value of the adhesion and cohesion POTS of asphalt after moisture-induced damage. (2) For asphalt A, asphalt B and their model asphalt, the POTS Ratio after conditioning time of 168 h is less than that after 1 h, especially for the model asphalt added to C20. This is because the specimen will be soaked in water for 48 h before the adhesion POTS test, and the 48 h after conditioning time 1 h can complete partial physical hardening, which improves the adhesion POTS. However, after a conditioning time of 168 h, the physical hardening was almost completed, and the subsequent 48 h immersion in water could not improve its adhesion POTS. (3) The POTS Ratio of high-wax asphalt B and its model asphalt is higher than that of the corresponding low-wax asphalt A and its model asphalt, which indicates that the higher the wax content of base asphalt, the lower the moisture sensitivity of itself and its model asphalt.

### 3.2. SFE Test

In order to verify the accuracy of the BBS test, this paper analyzes the effects of the molecular weight distribution and the branching of wax and asphaltene on the moisture sensitivity of asphalt, based on SFE. The solution process of the surface free-energy moisture sensitivity parameter ER of asphalt is as follows: Equations (8)–(10) can be derived from Equations (6) and (7). By placing the surface free-energy parameters of asphalt, aggregate and water into Equations (8)–(10), the cohesion work of the asphalt itself, the adhesion work of the asphalt–aggregate interface (dry condition) and the adhesion work of the asphalt–aggregate interface (wet condition) can be obtained. The ER can be obtained by substituting the adhesion work under dry and wet conditions into Equation (11). The larger the ER, the smaller the moisture sensitivity, and on the contrary, the greater the moisture sensitivity. The aggregate is granite and the surface free-energy parameters are shown in Table 3.

(6) $$∆G_{ij}=\gamma_{ij}-\gamma -\gamma$$

(7) $$∆G_{ikj}=\gamma_{ij}-\gamma_{ik}-\gamma_{jk}$$

In Equations (6) and (7), *G_ij_* is the interfacial binding energy between two-phase materials, *γ_ij_* is the interfacial energy of two-phase materials, *γ_i_* is the surface energy of substance *i*, *γ_j_* is the surface energy of *j*, and *G_ikj_* is the interfacial binding energy of three-phase materials.

(8) $$W_{AA}=2\gamma_{A}=2\sqrt{\gamma_{A}^{LW}}+4\sqrt{\gamma_{A}^{+}\gamma_{A}^{-}}$$

(9) $$W_{AG}=2\left(\sqrt{\gamma_{A}^{+}\gamma_{G}^{-}}+\sqrt{\gamma_{A}^{-}\gamma_{G}^{+}}+\sqrt{\gamma_{A}^{LW}\gamma_{G}^{LW}}\right)$$

(10) $$\begin{array}{ll} W_{AWG} & =2\left[\sqrt{\gamma_{A}^{LW}\gamma_{W}^{LW}}+\sqrt{\gamma_{G}^{LW}\gamma_{W}^{LW}}+\sqrt{\gamma_{W}^{+}}\left(\sqrt{\gamma_{A}^{-}}+\sqrt{\gamma_{G}^{-}}\right)+\sqrt{\gamma_{W}^{-}}\left(\sqrt{\gamma_{A}^{+}}+\sqrt{\gamma_{G}^{+}}\right)\right.\\ & \left.-2\sqrt{\gamma_{W}^{+}\gamma_{W}^{-}}-\sqrt{\gamma_{A}^{+}\gamma_{G}^{-}}-\sqrt{\gamma_{A}^{-}\gamma_{G}^{+}}-\sqrt{\gamma_{A}^{LW}\gamma_{G}^{LW}}-\gamma_{W}^{LW}\right] \end{array}$$

(11) $$ER=\left|\frac{W_{AG}}{W_{AWG}}\right|$$

In Equations (8)–(11), *W_AA’_*, *W_AG’_*, *W_AWG_* and ER are cohesion work, adhesion work in a dry condition, adhesion work in a wet condition and the moisture sensitivity index, respectively.

#### 3.2.1. Cohesion and Adhesion Work Analysis Based on SFE

**Figure 9:**
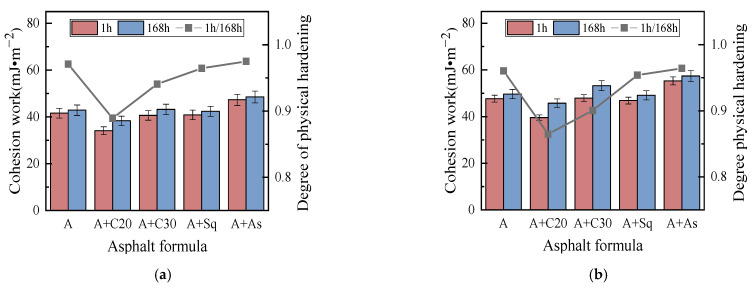
Cohesion work in dry conditions. (**a**) Asphalt A. (**b**) Asphalt B.

**Figure 10:**
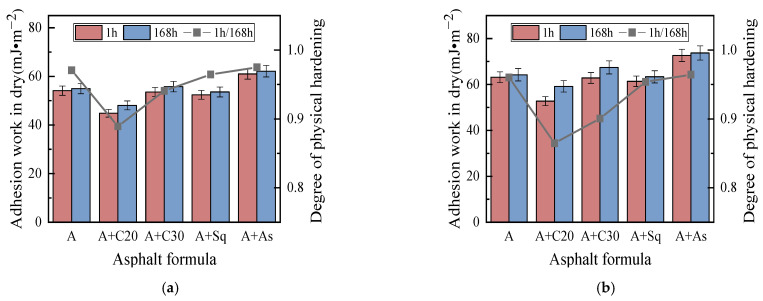
Adhesion work in dry conditions. (**a**) Asphalt A. (**b**) Asphalt B.

Figure 9 and Figure 10 show the cohesion work and adhesion work in dry conditions, respectively, which shows that: (1) when C20, C30/Sq, and As are added to asphalts A and B, respectively, the cohesion work and adhesion work in dry conditions are decreased, have no obvious effect and increase, respectively, and the effect is similar to the effect on the cohesion POTS (BBS parameter). (2) In dry conditions, the cohesion work and adhesion work of high-wax asphalt B and its model asphalt are larger than that of the corresponding low-wax asphalt A and its model asphalt, and the effect of the wax content of the base asphalt on the cohesion POTS (BBS parameter) is similar. (3) After the addition of C20 to asphalts A and B, the DPH is the largest, followed by C30, and Sq and As are not significantly influenced. The DPH is similar to that of the cohesion POTS (BBS parameter). The above conclusions show that the cohesion POTS of the BBS test and cohesion work of SFE verified the accuracy of each other’s test results. (4) In dry conditions, the adhesion work of all the model asphalts (including the base asphalt) is greater than that of the cohesion work, which explains, from the point of view of energy, that there is usually a cohesion failure rather than adhesion failure in the BBS test in dry conditions.

**Figure 11:**
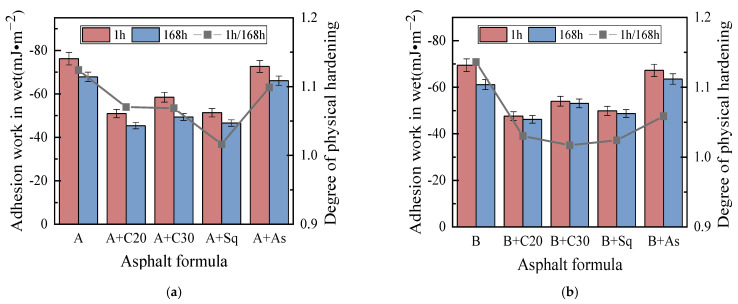
Adhesion work in wet conditions. (**a**) Asphalt A. (**b**) Asphalt B.

Figure 11 shows the adhesion work of asphalt samples in wet conditions, which shows that in wet conditions: (1) The adhesion work of all asphalt samples is a negative number, indicating that asphalt spalling from the aggregate surface occurs spontaneously in wet conditions, and the higher the value, the easier it is for asphalt to spontaneously peel off from the aggregate surface. (2) The addition of C20, C30 and Sq into A and B can reduce the absolute value of the adhesion work, however, the addition of As has no obvious effect on it. The results show that, in wet conditions, the addition of C20, C30 and Sq can alleviate the exfoliation of asphalt from the aggregate surface, while the addition of As has no obvious effect. (3) The addition of C20, C30, Sq and As to asphalts A and B, respectively, has no obvious regular effect on DPH, which is different from the effect on the cohesion work and adhesion work in dry conditions. A reasonable explanation is that the adhesion work in wet conditions is determined by the asphalt, aggregate and water— not only the asphalt and aggregate—which weakens the effect of the wax content on the physical hardening of asphalt. Existing studies believe that the effect of moisture on an asphalt–aggregate interface is extremely complex [4], and the specific reasons for the test results need to be further studied.

#### 3.2.2. Moisture Sensitivity Analysis Based on SFE

**Figure 12:**
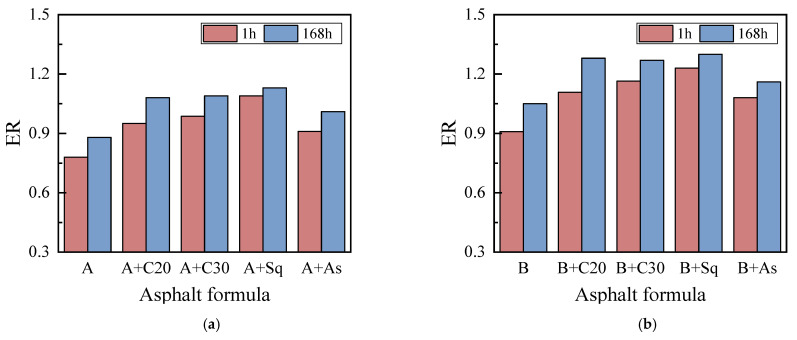
ER. (**a**) Asphalt A. (**b**) Asphalt B.

Figure 12 shows the effects of the molecular weight distribution and the branching of wax and asphaltene on the moisture sensitivity index, ER. It can be seen from the figure that: (1) The ER of high-wax asphalt B and its model asphalt is higher than that of the corresponding low-wax asphalt A and its model asphalt. In addition, the moisture sensitivity of asphalts A and B can be improved by adding C20, C30, Sq and As. The SFE test conclusion above is consistent with the BBS test conclusion. It is worth mentioning, that when As is added, the increase in the adhesion and cohesion work of asphalts A and B in dry conditions is stronger than when C20, C30 and Sq are added, however, the moisture sensitivity was lower than that of C20, C30 and Sq. This is because the absolute value of the adhesion work of the A/B + As condition, shown in Figure 11, is much larger than that of the alkane (C20, C30 and Sq) model of asphalt in wet conditions, resulting in a lower ER than the alkane-model asphalt. (2) The ER of all asphalt samples at a conditioning time of 168 h was larger than that at 1 h, which was contrary to the BBS test result. This is because the BBS test required soaking in water for 48 h before testing the adhesion POTS of the conditioning time of 1 h, during which, a part of the wax could be precipitated from the base asphalt, which was equivalent to the conditioning time of 49 h (1 h + 48 h). However, this phenomenon did not exist in the SFE test method. Therefore, when using the BBS test to characterize the moisture sensitivity of high-wax asphalt, it is recommended that the sample be placed for a certain amount of time and then tested after the physical hardening is stable.

### 3.3. Correlation between SFE and BBS

In order to study the correlation between the SFE and BBS tests in evaluating the moisture sensitivity of the molecular weight distribution and the branching of wax and asphaltene of the asphalt binder, two groups of parameters or indices, representing the same meaning in the two test methods, were analyzed. Among them, the cohesion POTS (BBS) and cohesion work (SFE) both characterize the degree of failure of the asphalt–asphalt interface, while the POTS ratio (BBS) and ER (SFE) both characterize the moisture sensitivity of asphalt.

**Figure 13:**
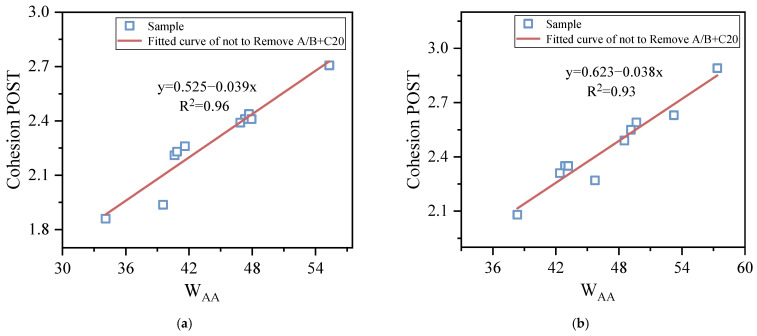
Correlation between cohesion POTS and cohesion work. (**a**) 1 h, (**b**) 168 h.

**Figure 14:**
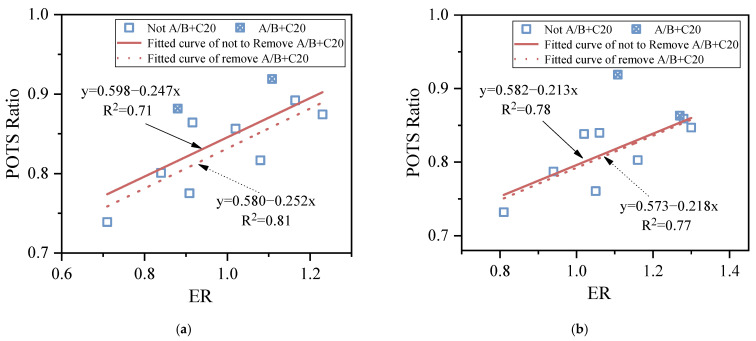
Correlation between POTS ratio and ER. (**a**) 1 h, (**b**) 168 h.

Figure 13 and Figure 14 show the correlation between the above two groups of parameters or indices, respectively. It can be seen that: (1) The correlation between the cohesion POTS and cohesion work for the conditioning times of 1 h and 168 h are 0.96 and 0.93, and the correlation coefficient between the POTS ratio and ER are 0.71 and 0.78, respectively. The results show that the cohesion POTS and cohesion work, POTS ratio and ER both have a good correlation, especially the former. (2) For the correlation between the POTS ratio and ER, if A + C20 and B + C20 were removed from the samples at the conditioning times of 1 h and 168 h, the correlation coefficients would change from 0.71 to 0.81 and 0.78 to 0.77, respectively. The results showed that the removal of A + C20 and B + C20 significantly increased their correlation at a conditioning time of 1h, while no significant difference was observed at a conditioning time of 168 h, as shown in Figure 14. This is because the BBS test method can easily lead to a large POTS ratio, especially for the asphalt samples with high physical hardening, while the SFE test method does not affect ER. The reasons for a higher POTS ratio of the BBS test have been described many times above, and will not be repeated here.

## 4. Conclusions

In this paper, the BBS and SFE tests are used to study the effects of the wax molecular weight distribution and branching on the moisture sensitivity of asphalt. The following conclusions can be drawn:

(1) The addition of n-eicosane, n-triacontane, squalane and asphaltene can reduce the moisture sensitivity of the base asphalt, but not necessarily improve its moisture-induced damage-resistance performance.
(2) For the cohesion POTS, regarding the cohesion and adhesion work in dry conditions, physical hardening has the greatest influence on n-eicosane, followed by n-triacontane. However, squalane and asphaltene have no obvious influence.
(3) The physical hardening effect of high-wax asphalt and its model asphalt is stronger than that of the corresponding low-wax asphalt and its model asphalt, and its moisture sensitivity is weaker than that of low-wax asphalt.
(4) Different molecular weight distributions and the branching of waxes will have different effects on the moisture sensibility of the asphalt binder.

## Figures and Tables

**Table 1:** Main technical indexes of asphalt A and B.

Asphalt Sample	Penetration, 25 °C (0.1 mm)	Ductility (mm)	Soften Point (°C)	Continuous PG (°C)	Wax Content * (%)	Asphaltene Content ** (%)
A	53	141	37	32.8–57.2	0.53	23.4
B	42	26	52	29.5–61.7	2.52	24.1

* The wax content was determined by method of distillation (EN 12606-1: 2015) [24]. ** The asphaltene content was determined according to ASTM D4124 [25].

**Table 2:** Main parameters of additives.

Parameters	Abbreviation	Formula	Purity	Density	Melting Point	Appearance
Eicosane	C20	C_20_H_42_	>99.0%	0.789 g/cm^3^	37 °C	Waxy crystals
Triacontane	C30	C_30_H_62_	>98.0%	0.810 g/cm^3^	66 °C	Waxy crystals
Squalane	Sq	C_30_H_62_	>99.0%	0.810 g/mL	−38 °C	Colorless liquid

**Table 3:** Surface free-energy parameters of chemical droplets and aggregate.

Liquids/Aggregate	*γ* ^+^	*γ* ^−^	*γ^AB^*	*γ^LW^*	*γ*
Distilled water (H_2_O)	25.5	25.5	51	21.8	72.8
Formamide (CH_3_NO)	2.28	39.6	19	39	58
Glycerol (C_3_H_8_O_3_)	3.92	57.4	30	34	64
Granite	9.87	0.56	4.70	45.69	50.39

## Data Availability

Not applicable.

## List of Notations and Abbreviations

BBS	binder bond strength
POTS	pull-off tensile strength
Adhesion POTS	POTS after moisture-induced damage
Cohesion POTS	POTS before moisture-induced damage
POTS Ratio	BBS moisture sensitivity index
SFE	surface free-energy
ER	SFE moisture sensitivity index
*γ*	surface free-energy
*γ^LW^*	dispersion component
*γ^AB^*	polarity component
*γ* ^+^	acid component
*γ* ^−^	basic component
*θ*	contact angle
*G* *_ij_*	interfacial binding energy between two-phase materials
*γ_ij_*	interfacial energy of two-phase materials
*γ_i_*	surface energy of substance *i*
*γ_j_*	the surface energy of j
*G* *_ikj_*	interfacial binding energy of three-phase materials
*W_AA_*	cohesion work
*W_AG_*	adhesion work
*W_AWG_*	adhesion work in wet condition
DPH	abbreviation of degree of physical hardening
*Value (1 h)*	moisture sensitivity parameter values of conditioning time 1 h
*Value (168 h)*	moisture sensitivity parameter values of conditioning time 168 h
